# The effect of an educational program on the knowledge and practices of diabetic patients regarding sharps waste disposal at home

**DOI:** 10.1038/s41598-024-81308-y

**Published:** 2024-12-23

**Authors:** Hossam Mohamed Hassan Soliman, Aleya Hanafy El-Zoka, Ebtisam Mohamed Fetohy, Mohamed Fakhry Hussein

**Affiliations:** 1 Department of Occupational Health and Industrial Medicine, High Institute of Public Health, Alexandria University, Alexandria, Egypt; 2 Health Administration and Behavioural Sciences Department, High Institute of Public Health, Alexandria University, Alexandria, Egypt

**Keywords:** Diabetes mellitus, Sharps disposal, Insulin syringes, Environmental program, Knowledge, Practice, Health care, Medical research

## Abstract

The disposal of sharp waste generated at home by diabetic patients poses a major public health problem. This study evaluated an educational program designed to improve patients’ knowledge and practices around sharps disposal. A non-equivalent quasi-experimental study was performed at a main diabetic center in Egypt on 100 participants divided into intervention and non-intervention groups. The effect of the interventional program was tested by the evaluation of the improvement in participants’ knowledge and practice after two and four months of the program. Initially, there were no differences in knowledge or practice scores between the groups. After the implementation of the program, there was a noticeable difference in subjects’ knowledge levels and scores between the two groups, with the intervention group significantly outperforming the non-intervention group (*p* < 0.001). There was a significant improvement in overall practice scores in the intervention group across post-intervention visits, and they registered a score of 14/16, which matches a good practice level, while in the non-intervention group, the score remained unchanged at 4.4/16, which corresponds to a poor practice level (*p* < 0.001). These findings suggest that educational programs can effectively increase diabetic patients’ knowledge and improve their sharps waste disposal practices.

*Clinical trial registration number*: PACTR202310841894237. Date: 05 October 2023, “retrospectively registered”.

**Supplementary Information:**

The online version contains supplementary material available at 10.1038/s41598-024-81308-y.

## Introduction

Diabetes mellitus (DM) is a chronic disease resulting from an inherited and acquired deficiency in the production of insulin by the pancreas (type 1 DM) or from the ineffectiveness of the insulin produced (type 2 DM). This deficiency results in elevated concentrations of glucose in the blood, which in turn damages many of the body’s systems, especially the blood vessels and nerves^1^.

More than 830 million persons are currently living with diabetes worldwide, which represents 12% of the world’s population. The Middle East and North Africa (MENA) region has the highest comparative prevalence of diabetes (18.1%) in people aged 20–79 years. The International Diabetes Federation (IDF) has listed Egypt among the world’s top 10 countries in terms of the number of patients with diabetes. The DM prevalence in Egypt has increased rapidly within a relatively short period, from approximately 4.4 million cases in 2007 to 10.9 million cases in 2021. Experts predict that this number will further escalate to 20 million by 2045^1,2^.

Diabetic patients, especially those with type 1 diabetes and some with type 2 diabetes, often require insulin injections at home. This involves at-home use of sharps, including insulin pens, syringes, and lancets, which generates sharp waste. Additionally, many diabetics rely on self-monitoring of blood glucose (SMBG) to maintain normal blood glucose levels, further contributing to sharp waste from self-checks. Given the significant number of diabetic patients, ensuring the safe disposal of these sharps is crucial^3,4^.

The disposal of generated sharps is a growing concern for public health, finances, and environmental safety. For the environment, improper disposal of sharps can contaminate soil and water with harmful pathogens and pose risks to both humans and animals through accidental injuries. While there’s extensive research on safe sharps disposal in hospitals, there’s a major gap in information about how diabetic patients dispose of sharps at home. Studies worldwide show that many diabetic patients don’t dispose of medical waste properly^5–9^. This improper disposal creates additional risks. In some parts of the world, people scavenge through waste disposal sites or sort hazardous medical waste from both healthcare facilities and home care by hand. This practice, particularly common in low- and middle-income countries, exposes waste handlers to a serious danger of needle-stick injuries (NSI) and infections from contaminated materials^7^.

Looking at the worldwide risk of infection, NSI can spread more than 15 viral diseases. The most significant ones are human immunodeficiency virus (HIV), hepatitis B virus (HBV), and hepatitis C virus (HCV), which have high seroconversion levels upon exposure to infected blood^3^. Furthermore, infectious agents that have the potential for transmission through unsafe injection practices, such as the Ebola virus, could spread easily^5^. Accidental NSI can have a major financial and emotional impact. The economic burden includes the cost of testing the injured person for infections, preventative medications, potential long-term treatment for blood-borne illnesses, staff replacement, counseling, and even legal fees^10–13^. Psychologically, NSI can lead to depression, anxiety, post-traumatic stress disorder (PTSD), insomnia, and panic attacks^10^.

In addition to the risk of NSI, improper disposal of sharps by diabetic patients contributes to significant plastic waste. This waste can be improperly incinerated, releasing harmful chemicals like Polychlorinated Biphenyls (PCBs), dioxins, and furans into the environment. These substances pose serious threats to both environmental and human health^14^.

There is growing evidence that diabetic patients actively participating in community programs for safe sharps disposal can significantly reduce public health risks^15,16^. Many developed countries, such as the United States and Australia, have implemented strict regulations for safe home sharps disposal. Instead of discarding sharps in the trash, several safe options are available, including designated drop-off sites, household hazardous waste collection programs, residential special waste pickup services, mail-back programs, syringe exchange programs, and home needle destruction devices. These policies apply to all patients with chronic diseases, including diabetics, who generate sharps waste through home care^15,16^. However, research also highlights a key challenge: a lack of awareness about the risks associated with improper sharps disposal, insufficient guidance from healthcare providers on safe disposal methods, and limited access to proper sharps disposal facilities for diabetic patients^5^.

While previous studies have shown that educational programs can improve diabetic patients’ knowledge about safe sharps disposal^17,18^, they haven’t necessarily translated into better disposal practices. This study aimed to evaluate the effectiveness of an educational intervention program, incorporating both traditional education and practical elements, in improving knowledge and practices related to safe sharps disposal among diabetic patients.

## Methodology

### Study setting

The researchers conducted the study between November 2022 and April 2023 at El-Horraya Polyclinic, Health Insurance Organization (HIO), Alexandria, Egypt.

### Study design

This is a quasi-experimental study, with unequal groups.

### Target population

Investigators recruited adult diabetic patients for this study who had been on regular insulin injections for more than a month, whether type 1 or type 2, and attended the El-Horraya polyclinic affiliated with the Egyptian HIO, Alexandria. Researchers excluded pregnant women with gestational diabetes.

### Sampling method and sample size

We used a systematic random sample. We selected every fourth patient who met the inclusion criteria until we reached the required sample size. We visited the diabetic center six days a week, dedicating Saturday, Monday, and Wednesday to selecting participants for the non-intervention group, and Sunday, Tuesday, and Thursday to selecting participants for the intervention group. We assigned participants to intervention and non-intervention groups based on their attendance time at the diabetic center to minimize potential information contamination.

According to a previous study^18^, the effect size (η^2^) of an environmental education program on the knowledge and practices of diabetic patients regarding sharp waste disposal at home was 0.318 (the mean difference in knowledge and practice scores before and after the intervention). Using an alpha error of 5% and a study power of 80%, the number of groups was 2 and the number of repetitions was 3 (we evaluated the participants through 3 visits: baseline, two months, and four months); the minimum required sample size was 54 diabetics, which was increased to 100 (50 intervention and 50 non-intervention) to account for potential dropouts or non-responses. Researchers calculated the sample size using G. Power software.

### Data collection methods and tools

We conducted in three phases as follows:

#### Phase I: a pre-intervention phase

##### A predesigned interview questionnaire

We developed an Arabic questionnaire about the disposal of sharps related to insulin therapy, adapting a questionnaire from the Alhazmi et al. (2022) study^19^. We translated the questionnaire to Arabic and back-translated it according to WHO guidelines. To guarantee the questionnaire’s validity and clarity, three professors (public health, health education, and internal medicine) reviewed it. They recommended adding a few new questions and refining existing ones. We then conducted a pilot study to identify and fix any problems with the questionnaire before using it in the main study. Finally, we tested the reliability of the revised questionnaire using Cronbach’s alpha test. This test yielded a score of 8.1, indicating the questionnaire has excellent internal consistency, meaning the questions all measure the same concept effectively.

The questionnaire had four main sections to collect data from both the intervention and non-intervention groups of diabetic patients. The first part asked about socio-demographic data (sex, age, residence, marital status, education, occupation, and crowding index). Researchers calculated the crowding index according to American Crowding Index guidelines, which consider crowding to occur when there is more than one person per room; severe crowding occurs if there are more than 1.5 persons per room (excluding bathrooms, balconies, porches, foyers, hallways, and half-rooms)^20,21^.

The second part covered the medical characteristics of diabetic patients, such as medical history, duration of diabetes, type of insulin injection, and duration of insulin use.

The third part of the questionnaire assessed knowledge through ten items covering three major concepts: proper syringe/needle use, hazards of improper disposal, and proper syringe/needle disposal. Participants received one point for each correct answer and zero points for incorrect answers. We converted their total score into a percentage (from 0 to 100%). We categorized the knowledge level scoring system into three levels: poor level (0 to less than 50%), fair level (50 to less than 70%), and good level (70–100%).

The fourth part assessed the practice through two subdivisions the first one (Proper Disposal Method) assessed whether participants disposed of sharps correctly. The recommended method was to return them in designated containers to healthcare facilities^18^. Participants received a score of 4 for proper disposal and 0 for improper disposal. The second subdivision (Knowledge Application) included ten multiple-choice questions (one correct answer each) on various topics related to sharps disposal, including different methods of disposal, using insulin pens and syringes, using a glucometer, needle stick injuries, and receiving advice regarding insulin injection device disposal. Each correct practice received 1 point, and incorrect answers received 0. A score of 0 to less than 5 was considered “poor practice,” a score of 5 to less than 7 was considered “fair practice,” and a score of 7 to 10 was considered “good practice”.

We calculated the overall practice score of the fourth part by combining the scores from the two subdivisions. This score ranged from 0 to 14. A score of 0–6 indicated poor practice, 7–10 indicated fair practice, and 11–14 indicated good practice.

The fifth part assessed the compliance of the intervention group with the program. We gave participants points based on their attendance at follow-up visits: 2 points for attending both visits, 1 point for attending one visit, and 0 points for no attendance.

The questionnaire is available in Supplementary File I.

### Pilot study

Researchers carried out a pilot study before implementing the actual study. It entailed pre-testing the questionnaire on five participants who were not included in the final analysis. Investigators carried out the pilot study to determine the validity of the tool, modify and adjust some questions, estimate the average time needed to obtain the required information, which was approximately 15 min, and identify obstacles that could be faced during the implementation of the study.

#### Phase II: intervention phase

The intervention group received the environmental educational program, whereas the non-intervention group did not receive any intervention.

##### Program description

Researchers conducted the intervention program through three educational sessions. It comprised two main components:

1. **Theoretical Component**: Through conducting three educational sessions to address the risks associated with improper sharp waste disposal, the environmental and health consequences, the benefits of safe disposal methods, and different modalities for safe disposal.
2. **Practical Component**: At the end of the third session, researchers conducted a demonstration to showcase the correct method of disposing of sharps into puncture-proof containers. We provided participants with free, puncture-resistant containers to safely dispose of sharp waste generated from diabetes management. We asked them to collect their used sharps in these containers and return them during follow-up visits to exchange for new ones.

To accommodate COVID-19 restrictions, we divided the 50 participants in the intervention group into 5 subgroups of 10. Each subgroup underwent three educational sessions over five weeks, with each session lasting 30–45 min. Educational methods included lectures and group discussions, audiovisual materials such as PowerPoint presentations and posters, and printed health education leaflets.

#### Phase III: post-intervention phase

Researchers assessed the impact of the program using the same interview questionnaire administered before the intervention. We compared the intervention and non-intervention groups at baseline, two months, and four months to evaluate the effectiveness of the educational program. Using the same questionnaire ensured reliable data collection and accurate evaluation of the program’s impact on knowledge and practices related to sharp waste disposal. Additionally, we monitored the percentage of returned sharp-disposal containers at two and four months post-intervention.

### Ethical considerations

We followed the Declaration of Helsinki, as well as any later modifications or equivalent ethical standards^22^. The researcher sought the approval of the Ethics Committee of the accredited institution for carrying out the research (High Institute of Public Health, Alexandria University, IRB number: 00013692; serial number: AU0922121329). The clinical trial registration number is PACTR202310841894237. We obtained informed consent from all participants before starting the research. We explained the study’s aim at the beginning, and participants could choose to participate or decline. Additionally, they had the option to leave the study at any point before it was finished. Participants received assurances that the information would only be used for research. Researchers kept participants’ responses anonymous and confidential. We educated the non-intervention group after the program ended.

### Statistical analysis and statistical tests

The researchers designed the questionnaire using the Epidemiological Information Package (Epi-Info) version 7 software to facilitate the extraction of data. Researchers collected all data from the interview questionnaires in an Excel spreadsheet, and then entered the data into R software version 4.2.2 (The R Project for Statistical Computing, Vienna, Austria) for analysis.

Frequencies and percentages were used to describe categorical data. The normality of continuous data was examined using the Shapiro-Wilk test, and it was found that they were all non-normally distributed. The median and interquartile range (IQR) were used to describe the non-normally distributed data. If applicable, statistical significance was determined using a p-value less than 0.05.

The chi-square test or Fisher’s exact test (as appropriate) was used to test associations between the categorical variables and the two study groups. We compared non-normally distributed variables between groups using the Mann-Whitney (U) test. We compared the proportions of the correct answers to the knowledge and practice questionnaire at each visit using the chi-square test. We used the Cochrane (Q) test to examine the effect of the intervention across time. Cochrane (Q) measured the proportion of patients returning used syringes or sharp waste containers to medical facilities across the three visits. Then, we used post-hoc McNemar tests to determine which pairwise proportions had significant differences. We used the Friedman test to compare knowledge and practice scores across the three visits. To identify specific differences between visits, we conducted pairwise comparisons using the Wilcoxon signed-ranks test with Bonferroni correction. Given the ordinal nature of the knowledge and practice levels (poor, fair, good), we used non-parametric rank tests to compare these levels between groups and over time, such as those used with the scores. Cronbach’s alpha coefficient measures the internal consistency, or reliability, of a set of survey items on a standardized 0–1 scale. A higher Cronbach’s alpha value indicates greater reliability of the survey instrument^23^. We used Kendall’s W coefficient test to assess the agreement between the rankings of knowledge scores and levels across the three visits for the two groups (intervention and non-intervention). This helps to determine if the intervention group consistently showed higher rankings compared to the non-intervention group over time^24^. We conducted both intent-to-treat and per-protocol analyses. The intent-to-treat analysis included all participants, regardless of program adherence or completion, while the per-protocol analysis included only participants who strictly adhered to the study protocol.

## Results

**Fig. 1:**
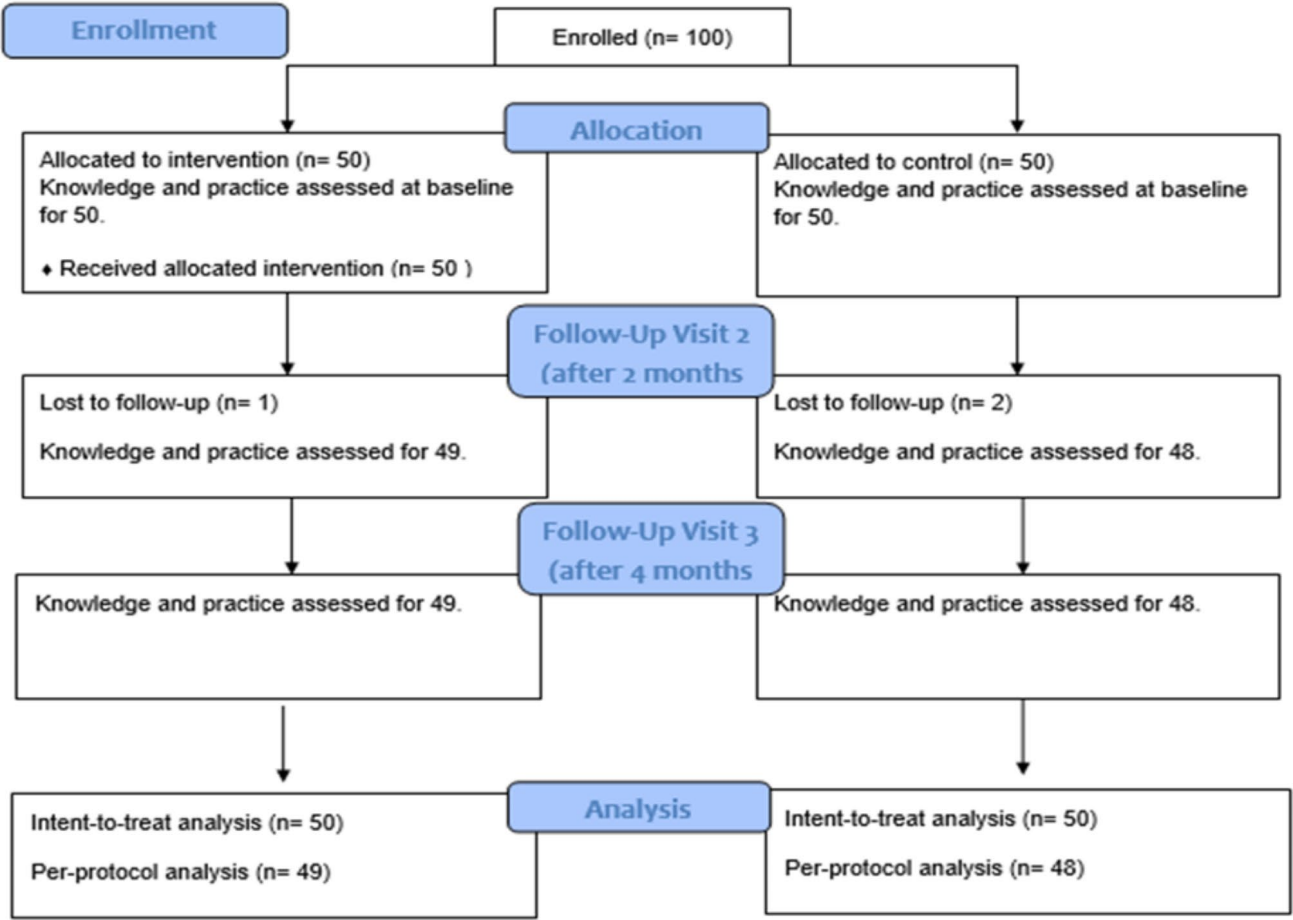
Flow diagram of participants in both the non-intervention and intervention groups (*n* = 100).

In this study, researchers enrolled 100 diabetic patients. We divided them into two groups: fifty patients were assigned to the intervention group, and the other fifty were assigned to the non-intervention group (Fig. 1). The follow-up for both groups lasted for four months, and overall adherence was excellent, with a 97% retention rate. Ninety-seven participants successfully completed the study. Of these, 49 participants were in the intervention group, and the other 48 participants were in the non-intervention group. The lost-to-follow-up participants were one in the intervention group due to failure to contact him and two participants in the non-intervention group due to refusal to come for follow-up. The researchers performed both statistical analyses, intention to treat (ITT), and per protocol (PP) analyses.

### Socio-demographic characteristics

Table 1 shows the distribution of participating patients according to socio-demographic characteristics. The majority of participants were aged 50 or older (78%), with a median age of 61 years. Most participants were married (77%). A significant proportion (28%) held graduate or postgraduate degrees. Concerning residence, 92% of the participants lived in urban areas, 8% in rural areas. The socio-demographic characteristics of the intervention and non-intervention groups did not differ significantly, as shown in Table 1.

**Table 1:** Distribution of the studied diabetic patients according to socio-demographic characteristics (*n* = 100).

Variable	Total (*N* = 100)	Intervention (*n* = 50)	Non-intervention (*n* = 50)	*p*-value
Gender				
Male	54 (54%)	28 (56%)	26 (52%)	0.420
Female	46 (46%)	22 (44%)	24 (48%)	
Age, Median (IQR)	61 (55–66)	62 (53–67)	60 (56–66)	0.812
Age categories				
30 to < 40	9 (9%)	4 (8%)	5 (10%)	0.933
40 to < 50	13 (13%)	7 (14%)	6 (12%)	
50 to < 60	22 (22%)	12 (24%)	10 (20%)	
60 or more	56 (56%)	27 (54%)	29 (58%)	
Marital status				
Widow	19 (19%)	9 (18%)	10 (20%)	1.000
Single	1 (1%)	1 (2%)	0 (0%)	
Married	77 (77%)	38 (76%)	39 (78%)	
Divorced	3 (3%)	2 (4%)	1 (2%)	
Occupation				
Without work (widow)	10 (10%)	5 (10%)	5 (10%)	0.914
Retired	50 (50%)	24 (48%)	26 (52%)	
Employee	40 (40%)	21 (42%)	19 (38%)	
Education				
Illiterate	3 (3%)	1 (2%)	2 (4%)	0.893
Read and write	7 (7%)	5 (10%)	2 (4%)	
Basic education	19 (19%)	8 (16%)	11 (22%)	
Diploma certificate	18 (18%)	10 (20%)	8 (16%)	
High diploma certificate	25 (25%)	12 (24%)	13 (26%)	
Graduate	24 (24%)	12 (24%)	12 (24%)	
Postgraduate	4 (4%)	2 (4%)	2 (4%)	
Residence area				
Civilized	92 (92%)	44 (88%)	48 (96%)	0.396
Rural	8 (8%)	6 (4%)	2 (4%)	

### Participants’ comorbidities

The current study demonstrated that one or more comorbid conditions affected 64% of the patients; hypertension and cardiac conditions were the most common. Our study concluded that hypertension was the most common comorbidity among diabetic patients (55%), and interestingly, 5% of participants suffered from the hepatitis C virus (HCV), as shown in Table 2.

**Table 2:** Distribution of the studied diabetic patients according to participants’ associated comorbidities (*n* = 100).

Variable	Total (*n* = 100)	Intervention (*n* = 50)	Non-intervention (*n* = 50)	*p*-value
Having at least one disease	64 (64%)	32 (64%)	32 (64%)	0.419
Hypertension	55 (55%)	26 (52%)	29 (58%)	0.419
Renal diseases	1 (1%)	0 (0%)	1 (2%)	1.000
Hepatic diseases	1 (1%)	0 (0%)	1 (2%)	1.000
Endocrine diseases	3 (3%)	1 (2%)	2 (4%)	1.000
Heart diseases	12 (12%)	7 (14%)	5 (10%)	0.758
Asthma	1 (1%)	1 (2%)	0 (0%)	1.000
Cholecystitis	1 (1%)	1 (2%)	0 (0%)	1.000
HCV	5 (5%)	2 (4%)	3 (6%)	1.000

### Descriptive data about diabetes, diabetes treatment characteristics, and medical history

Regarding the distribution of the participants according to history of diabetes, diabetes treatment characteristics, and medical history (Supplementary File II: Table 1; Fig. 1), the majority of the patients (67%) had diabetes for longer than 10 years, while 22% had used insulin for the same period. There was no significant difference between the intervention and non-intervention groups regarding the duration of diabetes and insulin use. The majority of participants used insulin pens (58%), and approximately three-fourths used two daily doses. The median number of syringes discarded each month per patient among syringe users was 10, whereas that of the disposed pen needles among pen users was 8. There were no significant differences in any of the medical traits between the intervention group and the non-intervention group of patients.

### Knowledge assessment

At the baseline visit (pre-intervention), the study findings demonstrated that there were no statistically significant differences in the knowledge levels and scores between the non-intervention and intervention groups, with nearly the same levels and a median knowledge percentage score of 60, which corresponds to the fair level category for both groups. Starting with the first post-intervention visit, after implantation of the program, there was a noticeable difference in subjects’ knowledge levels and scores between the two groups, with the intervention group significantly outperforming the non-intervention group in both measures as shown in Table 3.

**Table 3:** Distribution of the studied diabetic patients according to knowledge scores and categories pre- and post-intervention (*n* = 100).

Variable	Intent to treat analysis			Per protocol analysis		
	Intervention (*n* = 50)	Non-intervention (*n* = 50)	*p*-value	Intervention (*n* = 49)	Non-intervention (*n* = 48)	*p*-value
Baseline visit						
Score, Median (IQR)	60 (50–70)	60 (50–80)	0.123	60 (50–70)	60 (50–80)	0.111
Categories						
Poor	7 (14%)	6 (12%)	0.828	6 (12%)	5 (10%)	0.828
Fair	23 (46%)	21 (42%)		23 (47%)	20 (42%)	
Good	20 (40%)	23 (46%)		20 (41%)	23 (48%)	
Median (IQR)	2 (2–3)	2 (2–3)	0.551	2 (2–3)	2 (2–3)	0.497
First post-intervention visit						
Score, Median (IQR)	80 (80–90)	70 (60–90)	< 0.001*	80 (80–90)	75 (60–90)	< 0.001*
Categories						
Poor	1 (2%)	3 (6%)	< 0.001*	0 (0%)	1 (2%)	< 0.001*
Fair	0 (0%)	16 (32%)		0 (0%)	16 (33%)	
Good	49 (98%)	31 (62%)		49 (100%)	31 (65%)	
Median (IQR)	3 (3–3)	2 (2–3)	< 0.001*	3 (3–3)	3 (2–3)	< 0.001*
Second post-intervention visit						
Score, Median (IQR)	80 (80–90)	70 (60–80)	0.002*	80 (80–90)	70 (60- 82.5)	0.002*
Categories						
Poor	1 (2%)	5 (10%)	< 0.001*	0 (0%)	3 (6%)	< 0.001*
Fair	1 (2%)	13 (26%)		1 (2%)	13 (27%)	
Good	48 (96%)	32 (64%)		48 (98%)	32 (67%)	
Median (IQR)	3 (3–3)	2 (2–3)	< 0.001*	3 (3–3)	3 (2–3)	< 0.001*

IQR: Interquartile Range *: significant result *p* < 0.05.

The first post-intervention visit results showed that the participants of the intervention group had a higher median knowledge percentage score of 80 (80–90), which indicated a good knowledge level in both analyses, as compared to the non-intervention group with median percentage scores of 70 (60–90) and 75 (60–90) according to intent-to-treat analysis and per protocol analyses, respectively, and the difference reached statistical significance (*p* < 0.001). At the second post-intervention visit, we identified a higher median knowledge score of 80 (80–90) in the intervention group than in the non-intervention group, which was 70 (60–90) according to both analyses (*p* < 0.001), and the difference was statistically significant, as illustrated in Table 3.

Detailed changes in knowledge percentage scores and levels are available in Supplementary File II: Figures 3 and 4. Friedman test values and effect sizes (Kendall W) for the repeated measures of the knowledge scores and levels are available in (Supplementary File II: Table 2) which measured the change in knowledge regarding sharps disposal before and after the intervention. Pairwise comparisons in knowledge scores and levels between visits are available in (Supplementary File II: Table 3).

### Subdivision one of the practice assessment

**Fig. 2:**
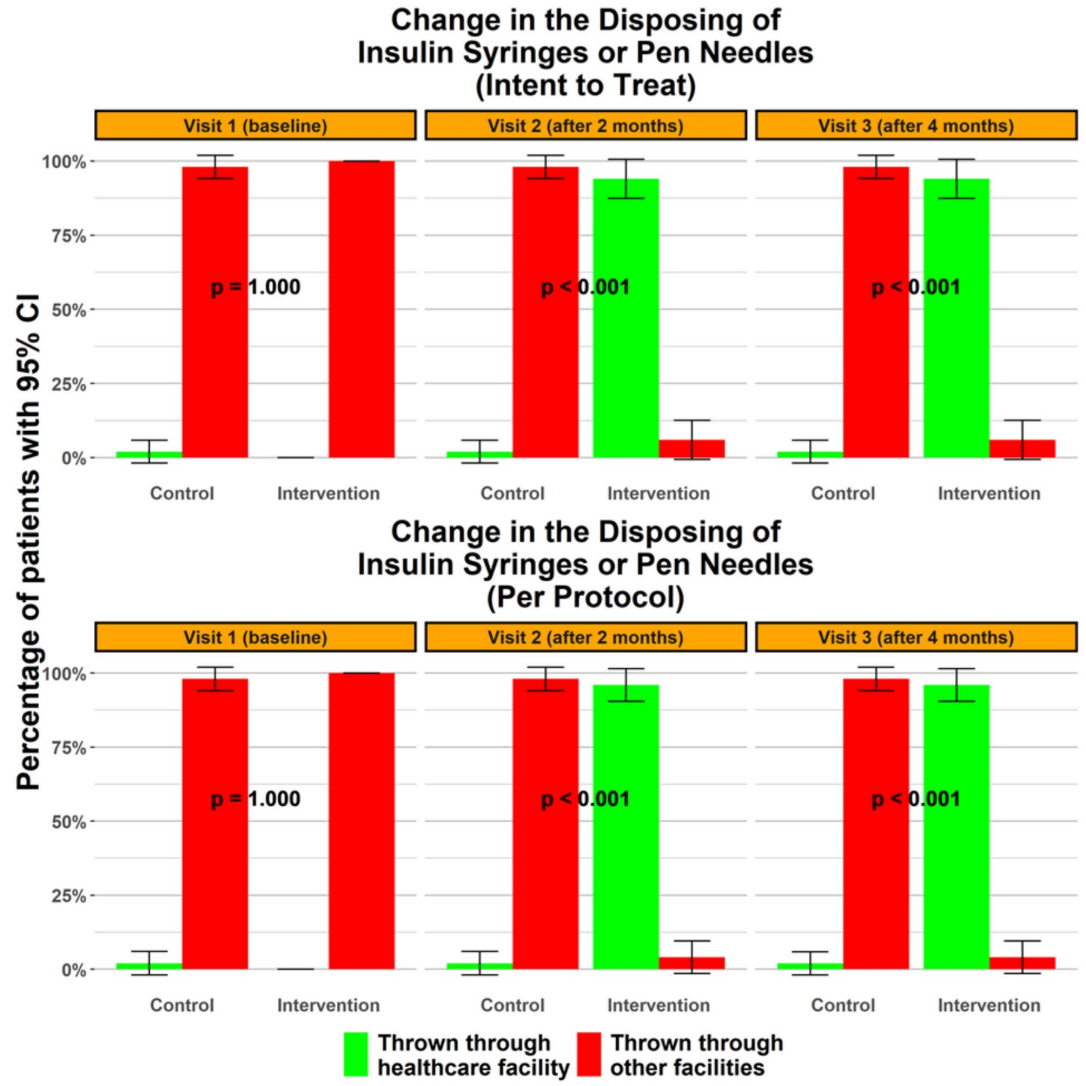
Change in the proportion disposing of insulin syringes or pen needles at health care facilities over visits.

As indicated in Fig. 2 and Supplementary File II: Table 4, the proportion of individuals who disposed of their insulin syringes or pen needles at medical facilities began to differ significantly from the first post-intervention visit, with the intervention group consistently surpassing the non-intervention group (*p* ≤ 0.001). Tables 5 and 6 in supplementary file II show detailed statistical tests for the proper disposal of insulin syringes or pen needles at health care facilities.

### Subdivision two of the practice assessment

**Fig. 3:**
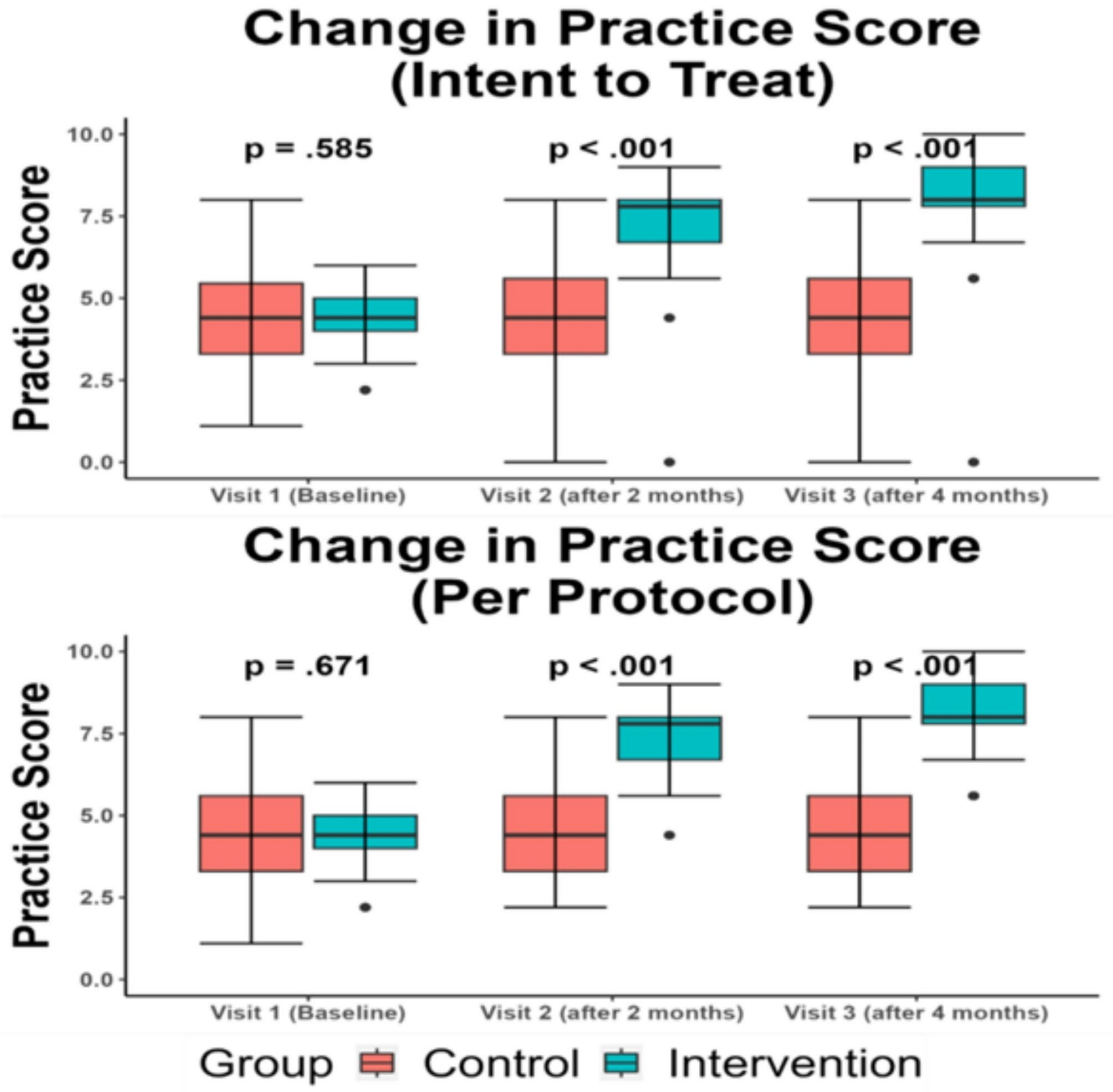
Change in practice scores through the visits.

**Fig. 4:**
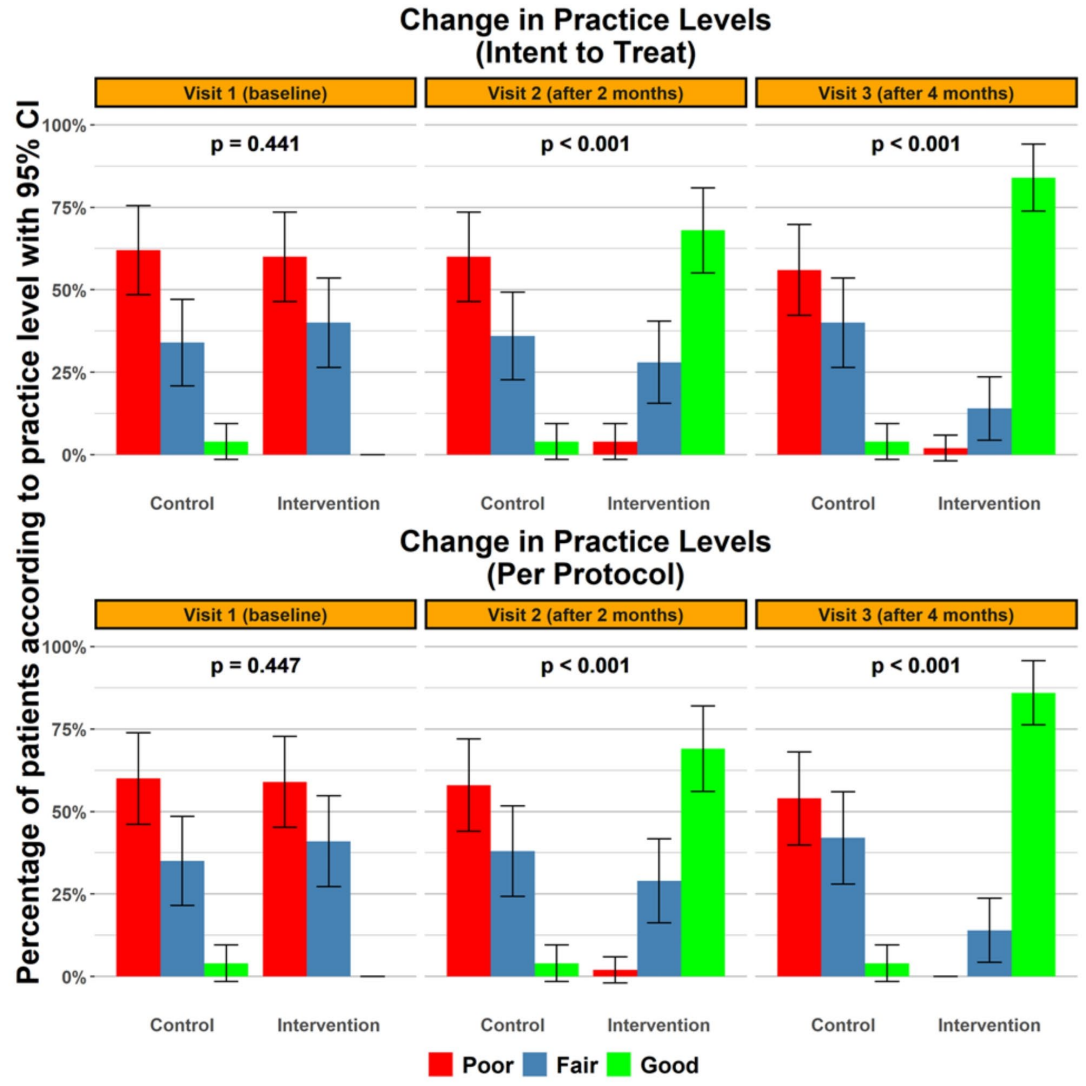
Change in practice levels through the visits.

The practice levels and scores of both groups were almost the same at the baseline visit, which corresponds to the poor practice scale. At the subsequent two visits, there were noticeable differences in participants’ practice levels and scores, with the intervention group significantly outperforming the non-intervention group. The first post-intervention visit results showed that the participants of the intervention group had a higher median practice score of 7.8 (6.7-8), which corresponds to good clinical practice in both analyses, compared to that of the non-intervention group, which was 4.4 (3.3–5.6) and 4.4 (3.3–5.5) according to intent-to-treat analysis and per-protocol analysis, respectively, and the difference reached statistical significance (*p* < 0.001). Likewise, at the second post-intervention visit, we identified a nearly double median knowledge score of 8 (7.8-9) in the intervention group as compared to the non-intervention group score of 4.4 (3.3–5.6) (*p* < 0.001), as shown in Fig. 3. Similar results were found regarding the practice levels with a statistically significant difference between the two groups (*p* < 0.001), which are highlighted in Fig. 4. Details of practice scores and categories pre- and post-intervention are available in Supplementary File II: Table 7.

### Overall practice assessment

At the baseline visit, there was no statistically significant difference between the intervention and non-intervention groups regarding the median overall practice scores, which recorded 4.4/14, which corresponds to a poor practice level. At the end of the program, there was a statistically significant difference between groups in the median of the participants’ overall practice scores, with the intervention group significantly outperforming the non-intervention group and registering a score of 12, which matched the good practice level, while the non-intervention group stopped unchanged at 4.4. The within-group analysis showed a statistically significant increment in the overall practice scores among the intervention group at the end of the program compared to baseline in the intent-to-treat (V = 3, *p* < 0.001) and the per-protocol analyses (V = 0, *p* < 0.001). These results reflected the positive impact of the program on practice (Table 4).

**Table 4:** Overall practice scores at baseline and end of the program.

Variable	Intent to treat analysis			Per protocol analysis		
	Intervention (*n* = 50)	Non-intervention (*n* = 50)	Between group *p*-value	Intervention (*n* = 49)	Non-intervention (*n* = 48)	Between group *p*-value
Overall practice score at baseline: Median (IQR)	4.4 (4–5)	4.4 (3.3–5.5)	0.585	4.4 (4–5)	4.4 (3.3–5.6)	0.671
Overall practice score at end of program: Median (IQR)	12 (11.5–13)	4.4 (3.3–5.6)	< 0.001*	12 (11.5–13)	4.4 (3.3–5.6)	< 0.001*
Within group p-value	< 0.001*	0.844		< 0.001*	0.185	

IQR: Interquartile Range * Significant results *p* < 0.05.

### Compliance to the intervention program

Regarding the intervention group, as shown in Supplementary File II: Fig. 5, there was a significant percentage of respondents who adhered to and completed all sessions of the program, which recorded 96% at the first (visit 2) and second (visit 3) post-intervention visits (one missing participant due to failure to contact him and another participant refused to bring the sharps container back to the clinic).

## Discussion

The current study assessed the impact of an intervention program on diabetic patients’ knowledge and practices of safe sharps disposal at home. Before the program, both intervention and non-intervention groups exhibited fair knowledge levels and poor disposal practices. The program significantly improved participants’ knowledge and practices, where participants were provided with free sharps-disposal containers and demonstrated a substantial increase in proper disposal at healthcare facilities.

While the present study explored the prevalence of comorbid conditions among participants, it demonstrated that 64% of participants had at least one comorbid condition, with hypertension and cardiac conditions being the most prevalent. In particular, hypertension was identified as the most common comorbidity, affecting 55% of diabetic patients, aligning with the findings of an Ethiopian systematic review and meta-analysis^25^.

The present research demonstrated that the majority of participants (58%) used insulin pens for injections, a higher proportion than the 17.8% reported in a Pakistani study^26^. Additionally, around three-quarters of the participants injected insulin twice daily. This aligns with a study by Hassan et al. (2021), where 73.2% of participants followed a similar regimen^27^.

Furthermore, our study found that syringe users discarded an average of 10 syringes per month, while pen users discarded an average of 8 pen needles per month. This aligns with research by Basazn et al. (2016), who stated that about 87% of diabetic patients disposed of 0–14 syringes in a week^28^. These findings support the hypothesis that an enormous number of discarded sharps will be generated in community settings aligned with the growing number of diabetic patients in Egypt. This highlights the urgent need for effective solutions to manage this growing problem. We need comprehensive and innovative programs to ensure that medical sharps related to diabetes are disposed of safely and properly in local communities.

Before the educational program (baseline visit), both the intervention and non-intervention groups had similar knowledge levels about sharps disposal, with a median score of around 60%. However, after the program (first post-intervention visit), the participants who received the educational intervention showed a significant improvement in their knowledge. Their median score jumped to 80%, indicating a good level of understanding, compared to the non-intervention group. This positive change was statistically significant (*p* < 0.001). The knowledge gap between the two groups persisted even at the second post-intervention visit. The persistence of good knowledge over time indicates the full understanding and retention of information by patients.

These results are in the same vein as those of an Egyptian study conducted at Banha University, which illustrated that there was an increase in the mean knowledge score from 57.2 ± 27.8 (pre-intervention) to 85.2 ± 16.2 after the intervention and that the proportion of good knowledge scores increased from 65.2 to 85.4%^17^. These results were supported by a study conducted in Indonesia that concluded that education received from healthcare providers significantly improved the knowledge level of patients^29^.

Interestingly, the non-intervention group also showed a small but statistically significant increase in knowledge scores at both follow-up visits compared to the baseline. This suggests that simply bringing attention to the issue of sharps disposal may have motivated participants to seek out accurate information on their own, even though they weren’t directly involved in the educational program.

Our study showed a clear difference between the intervention and non-intervention groups regarding safe sharps disposal practices (disposing of sharps at healthcare facilities) after the educational program. At the first and second follow-up visits, a significantly higher proportion of participants in the intervention group disposed of sharps safely compared to the non-intervention group. We did not observe a significant difference between the groups at the baseline visit. These results were consistent with another study from the USA. That study also reported a significant decrease in unsafe sharps disposal practices after an educational program. The percentage of participants who disposed of sharps unsafely dropped from 39.6 to 10.4%^30^.

In the same way, there were comparable results in the Malaysian study, which clarified that there was a significant increase in the proportion of participants who practiced the proper sharps disposal method in the intervention group from 1.5% at the baseline visit to 16% and 50% at subsequent post-intervention visits, while the proportion remained constant at the three visits in the non-intervention group at 1.5%^18^.

A previous study by El Gendi et al. (2017) found that even after educational programs on insulin device disposal, only 18% of participants achieved good practice scores^17^. This suggests that education alone may not be enough. Our study had a unique intervention because it combined education with a free sharps-disposal program. This might explain the significant improvement in safe sharps disposal practices compared to other studies. While other programs focus solely on raising awareness, ours provided a practical solution alongside education.

This high percentage of compliance in our study suggests that the majority of insulin-treated diabetic patients are ready to participate in any structured community sharps disposal program, particularly when it is implemented in an accessible and free manner. These results have crucial implications for healthcare providers and public health officials. The success of the environmental education program underscores the need for targeted interventions to enhance patient knowledge and safe sharps disposal practices. By offering accessible disposal options and raising awareness, healthcare facilities can significantly contribute to preventing accidental needle-stick injuries and reducing the risk of infectious disease transmission^7,15,16^.

### Study limitations and strengths

The research had some limitations. First, the study design was based on interview-reported assessments, which resulted in interview bias, which means that the interviewer’s personal opinions or beliefs could influence the assessment of an interviewee, leading to unfair judgments. To minimize this, we used a structured interview questionnaire with standardized questions for all candidates, and we asked the questions in the same manner. Besides, we conducted the interviews in a controlled setting, ensuring consistent environmental conditions for all participants. The controlled setting means that the environmental conditions, such as temperature, humidity, and noise levels, were carefully regulated to create a comfortable and conducive environment for the participants. This approach aimed to minimize external distractions and biases that could potentially influence the responses. The second limitation was the possibility of self-reporting bias, as participants might not always report their actions accurately. To address this, we observed participants disposing of sharps at a healthcare facility. This provided a more reliable assessment of their disposal practices compared to solely relying on their self-reported scores. Another limitation is attrition bias, which occurs when participants drop out of a study. To minimize this, we used a technique called intention-to-treat analysis. This approach includes data from all participants, even those who didn’t complete the study. Additionally, we employed a per-protocol (PP) analysis, which only considers data from participants who fully adhered to the program. Fortunately, the high retention rate of both methods yielded similar results. Finally, the quasi-experimental study design, which lacks randomization, may limit the strength of causal inferences.

Despite these limitations, this study provides valuable local data and demonstrates the effectiveness of a combined educational and practical intervention, including the distribution of free sharp-disposal containers. This information can be used by policymakers and authorities to develop solutions for safe sharps disposal at home. The present study offers two distinctive features. First, it integrates a practical component (supplying the intervention group with free sharp-disposal containers) with the educational program. Second, it achieved a high participant retention rate, suggesting similar programs could be successful in the future. Furthermore, even without the intervention, patients in the non-intervention group showed an increased awareness of the issue, highlighting their willingness to participate in community sharps disposal programs.

## Conclusion

The present study was designed to determine the effect of an environmental educational program on the knowledge and practices of diabetic patients regarding sharps waste disposal at home. The results of the current study show that the majority of patients had a fair knowledge level and did not practice acceptable sharps disposal methods before the implementation of the environmental educational program in both non-intervention and intervention groups.

The study revealed that the program was effective in improving the knowledge and practices of the participants regarding sharps waste. The most obvious finding to emerge from this study is that there was a marked improvement in the total practice score and proportion of the participants who disposed of sharps properly at healthcare facilities within the intervention group (as we supplied them by free sharps disposal containers) rather than the non-intervention group. The significant percentage of respondents who adhered to and completed all program sessions and the positive outcome of the current program suggest the necessity to implement a proper community sharps disposal program immediately, which is simple, convenient, and free of charge to promote the safety of the community. Advanced research will be required in the future to develop low-cost green diabetic programs to meet socio-cultural behaviors.

## Electronic supplementary material

Below is the link to the electronic supplementary material.

Supplementary Material 1

Supplementary Material 2

Supplementary Material 3

## Data Availability

The datasets used and analyzed during the current study are available from the corresponding author upon reasonable request.
