# Supplementary material for: The effect of an educational program on the knowledge and practices of diabetic patients regarding sharps waste disposal at home
Source: Sci Rep. 2024 Dec 23;14:30590. doi: 10.1038/s41598-024-81308-y (PMC11666584; doi:10.1038/s41598-024-81308-y)
Supplement: Supplementary file 1 — Supplementary Material 1 [file 41598_2024_81308_MOESM1_ESM.pdf]

Supplementary File I

The effect of an educational program on the knowledge and practices of diabetic patients regarding sharps waste disposal at home

Hossam Mohamed Hassan Soliman, Aleya Hanafy El-Zoka, Ebtisam Mohamed Fetohy, Mohamed Fakhry Hussein

Questionnaire on Effectiveness of Environmental Educational Program for diabetic patients on Sharps Disposal at home

Intervention group / Non-intervention group **ID:**

Baseline visit date:

First post-intervention visit: **Second post-intervention visit:**

Diabetic center: El-Horraya

A- Personal Data

**Serial Number**	
**Age**	**………………………. years**
Birth Date	
**Sex**	☐ Male ☐ Female
**Civil status**	☐ Single<br>☐ Married<br>☐ Divorced<br>☐ Widowed
**Employment**	☐ Employed<br>☐ Not Employed (widow)<br>☐ Self-employed<br>☐ Retired
**Highest educational attainment**	☐ Illiterate<br>☐ Primary school graduate<br>☐ Intermediate education graduate<br>☐ Institute graduate<br>☐ Graduate<br>☐ Postgraduate (MS, PhD, MBA, MD, etc.)
**Telephone / Cellphone Number**	
**Residence area**	☐ Civilized area<br>☐ Rural area<br>☐ Slum area

**Housing**	☐ Independence<br>☐ Shared with others
**Rooms number**	………………………room
**Number of bedrooms**	………………………room
**Number of persons**	…………………………..person
**Number of children**	……………………………. child

B- Second Part- Medical Data

**Please answer the following questions.**	
How long have you been diagnosed with diabetes?	
How many years have you been using insulin?	
What kind of insulin have you used?	☐ Insulin bottle (vial)<br>☐ Insulin pen<br>☐ Both insulin bottle and pen<br>☐ Insulin pump
How many insulin doses did you take daily?	…………………………..dose
How many insulin syringes do you dispose of monthly?	…………………………syringe
How many insulin pen needles did you dispose of monthly?	………………………. pen needle
Did you suffer from any other medical disease?	0 No<br>1 Hypertension<br>2 Heart disease<br>3 Liver disease<br>4 Kidney disease<br>5 Neural disease<br>6 Blood disease<br>7 Glandular disease<br>8 Eye disease<br>9 Others (specify)…………….

Third part - Questions assessing knowledge

1- If you inject insulin while outside the house, what should you do with the used insulin syringe?

A. Throw them in the nearest garbage can

B. Flush them down a toilet

C. Bring them back home

D. Throw them in the street

2- Which of the following is the best way to dispose of used insulin needles and syringes at home?

A. Flush them down the toilet

B. Bury them in the ground in your backyard

C. Place them in a puncture-resistant container and dispose with household waste

D. Burn them in your backyard

3- What is the best container for storing used insulin syringes or needles safely?

A. Plastic bottle

B. Glass bottle

C. Plastic bag

D. Metal can with no cover

4- What should you do before throwing away your used insulin syringe?

A. Pull out the needle using pliers

B. Cover the needle with the cap

C. Bend the needle

D. Cut the needle with scissors

5- On the country level used insulin syringes are best

A. Recycled like newspaper and bottles

B. Burned in the backyard

C. Given to other diabetics who use insulin so they can reuse them D. Given to the garbage man for disposal

D. Disposed of through community safe sharps disposal program

6- You plan to reuse your insulin syringe. What should you do after using it for the first time?

A. Put the cap back on

B. Clean the needle with alcohol

C. Sterilize the needle with a flame

D. Leave it as it is without cap

7- While injecting insulin, the insulin syringe slips out of your hand and falls to the ground. What should you do?

A. Use the syringe that fell but clean it with alcohol first

B. Wipe the syringe with a clean cloth then use it

C. Throw away the syringe and use a new one

D. Just use the syringe like nothing happened

8- Which infectious disease can you get after being accidentally pricked by a syringe used by another person?

A. Malaria

B. Tetanus

C. Cancer

D. Hepatitis

9- Who of the following people in the community is at greatest risk of getting accidentally pricked by used insulin syringes?

A. Garbage scavengers

B. Security officer

C. Teacher

D. Street vendor

10 Where is the best place to store your insulin syringes?

A. In the refrigerator, along with your insulin

B. In a high cabinet that is hard to reach

C. In a hidden place like underneath your clothes

D. In a drawer without a lock

Fourth part: Questions assessing practice

<u>Subdivision 1:</u>

Which of the following are the methods you use in disposing of your used insulin syringes?

- ☐ Thrown through health care facility.
- ☐ Thrown through other ways.

<u>Subdivision 2:</u>

1- Do you bend your insulin syringe needles before disposing of them?

- ☐ No
- ☐ Yes

2- Where do you place your used insulin syringes just before disposing them?

a. No container used

b. Plastic bag

c. Glass bottle

d. Uncovered plastic, cartoon, or metal container

e. Heavy duty and puncture resistant capped plastic or metal container

3- Who of the following has given you advice on how to dispose of your used insulin syringes?

a. Health care worker

b. Friends

c. Family members

d. None

4- How do you dispose of your used insulin syringes when you are outside the home?

- ☐ I bring them home
- ☐ I throw them in any available trash can
- ☐ I throw them in street
- ☐ I gave them to garbage collector

5- Aside from yourself, has anyone else been accidentally injured by your insulin syringes?

a. No

b. Yes

6- Did you share your insulin syringes or needles with others?

a. No

b. Yes

7- Did you use BGM device?

a. No

b. Yes

8- If yes, how did you dispose of your lancets?

- ☐ Thrown directly into the garbage can mixed with the household trash
- ☐ Thrown through health care facility.
- ☐ Thrown into toilet
- ☐ Thrown in street
- ☐ Not applied

9- Did you use a single syringe or pen needle more than once?

a. No

b. Yes

10- Did you keep your insulin syringes and pen away from children?

a. No

b. Yes

Fifth part: Compliance with the intervention program

The compliance of the participant in intervention group with the program

a. Completing both follow-up visits
b. Attended one visit
c. No attendance of any follow-up visits
