# Supplementary material for: The effect of an educational program on the knowledge and practices of diabetic patients regarding sharps waste disposal at home
Source: Sci Rep. 2024 Dec 23;14:30590. doi: 10.1038/s41598-024-81308-y (PMC11666584; doi:10.1038/s41598-024-81308-y)
Supplement: Supplementary file 2 — Supplementary Material 2 [file 41598_2024_81308_MOESM2_ESM.pdf]

Supplementary File II

The effect of an educational program on the knowledge and practices of diabetic patients regarding sharps waste disposal at home

Hossam Mohamed Hassan Soliman, Aleya Hanafy El-Zoka, Ebtisam Mohamed Fetohy, Mohamed Fakhry Hussein

Table (1): The percentage of the studied diabetic patients according to history of diabetes and diabetes treatment characteristics (n =100)

Variable	Total (n =100)	Intervention (n =50)	Non-intervention (n =50)	P
**Diabetes duration in years: Median (IQR)**	15 (10-20)	16 (10-20)	14 (9-20)	0.774
**Diabetes duration categories**				0.866
Less than 5 years	11 (11%)	6 (12%)	5 (10%)	
5 to 10 years	22 (22%)	10 (20%)	12 (24%)	
More than 10 years	67 (67%)	34 (68%)	33 (66%)	
**Insulin duration: Median (IQR)**	6 (3-10)	7 (3-10)	6 (3-10)	.912
**Insulin use duration categories**				0.975
Less than 5 years	37 (37%)	19 (38%)	18 (36%)	
5 to 10 years	41 (41%)	20 (40%)	21 (42%)	
More than 10 years	22 (22%)	11 (22%)	11 (22%)	
**Insulin type**				0.208
Syringe	36 (36%)	14 (28%)	22 (44%)	
Pen	58 (58%)	32 (64%)	26 (52%)	
Syringe and pen	6 (6%)	4 (8%)	2 (4%)	
**Daily injections: Median (IQR)**	2 (2-2)	2 (2-2)	2 (2-2)	0.562
**Number of daily injections**				0.496
1	15 (15%)	10 (20%)	5 (10%)	
2	73 (73%)	33 (66%)	40 (80%)	
3	9 (9%)	5 (10%)	4 (8%)	
4	3 (3%)	2 (4%)	1 (2%)	
**Number of discarded syringes per month: Median (IQR)**	10 (6-26)	13 (5-30)	8 (7-15)	0.690
**Number of discarded pen needles per month: Median (IQR)**	8 (5-10)	10 (6-15)	6 (4-10)	0.131

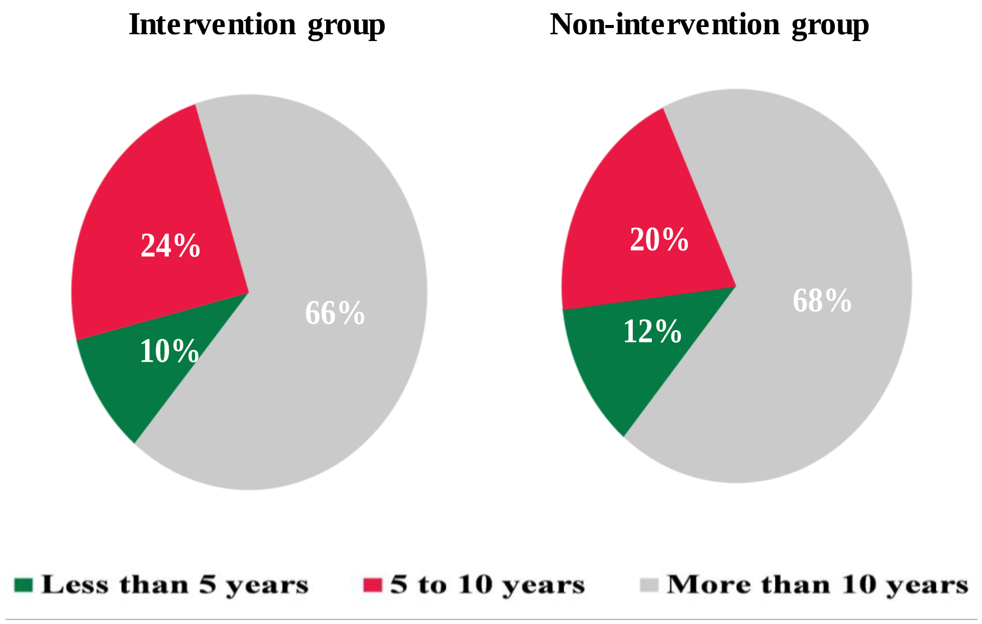

Figure (1): Duration of diabetes in the intervention and non-intervention groups

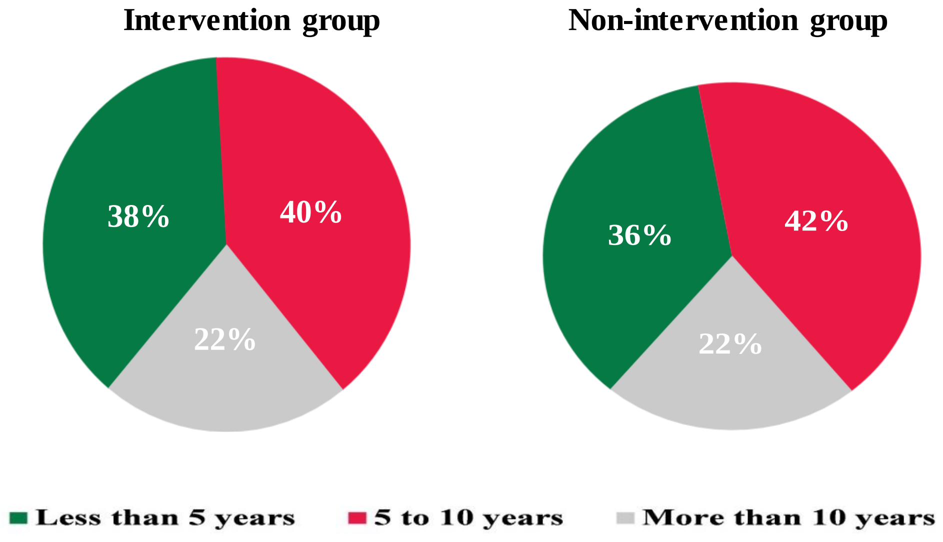

Figure (2): Duration of insulin usage in the intervention and non-intervention group

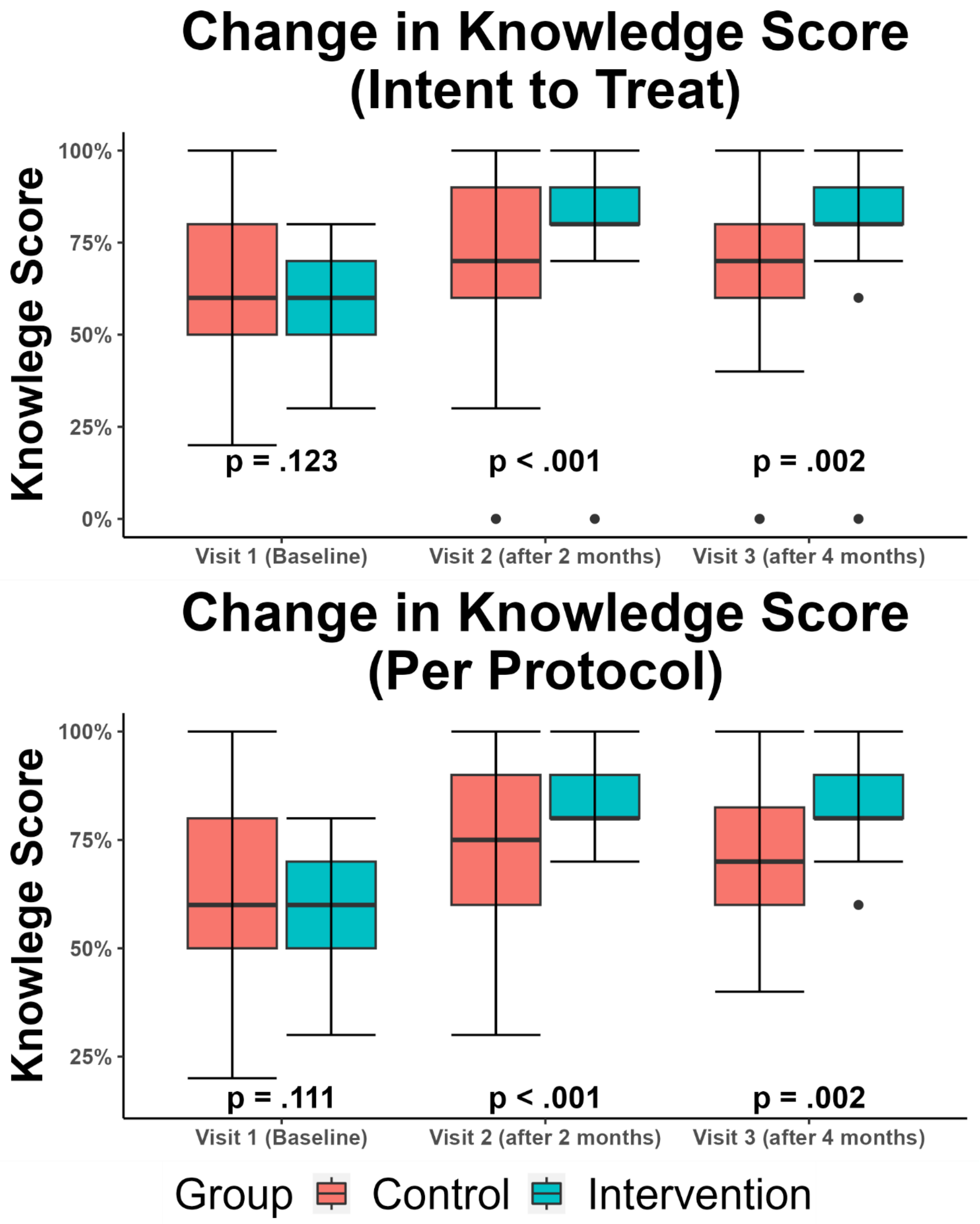

Figure (3): Change in knowledge scores

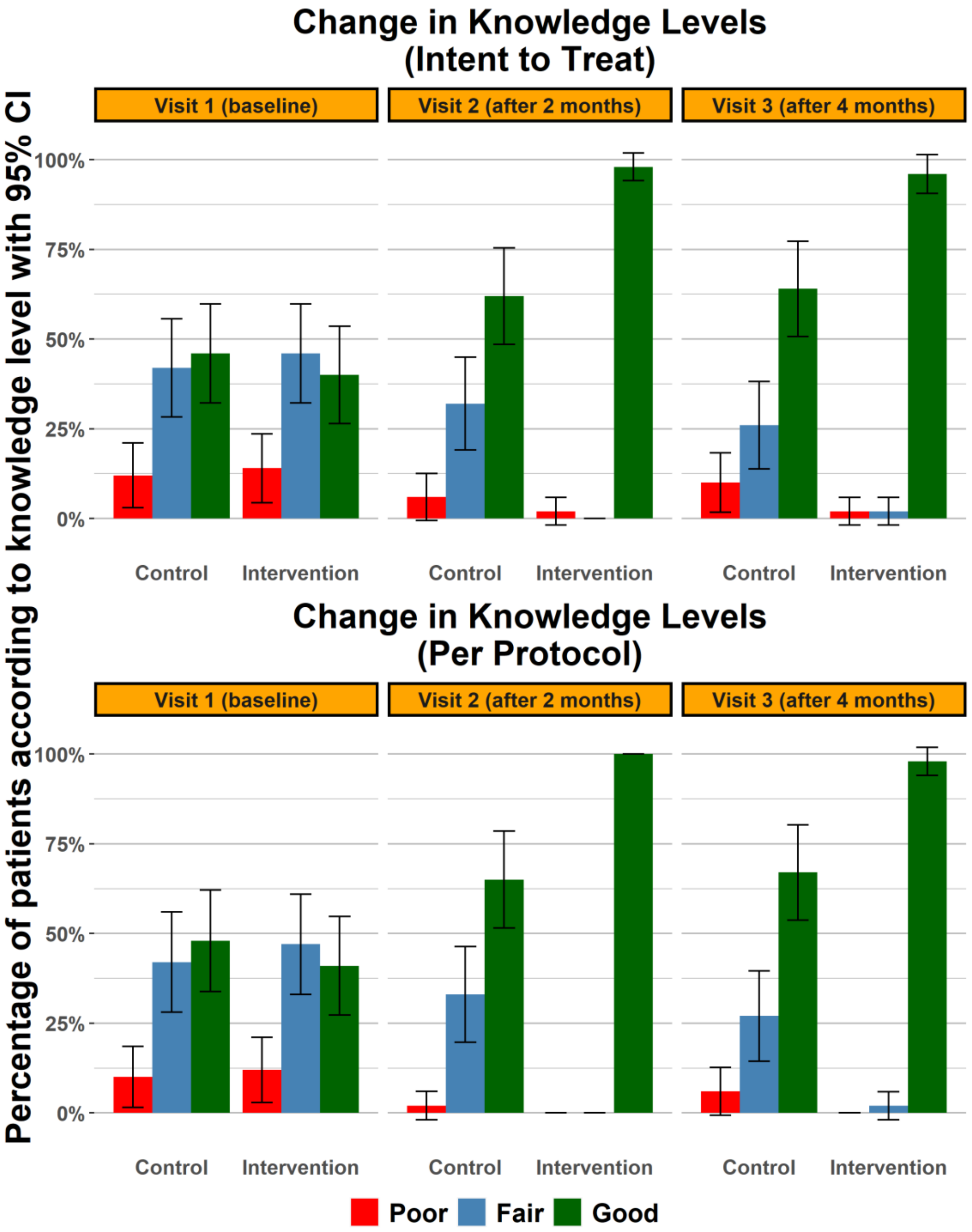

Figure (4): Change in knowledge levels

Table (2): Friedman test χ^2 values and effect sizes (Kendall W) for the repeated measures of the knowledge scores and levels (n =100)

Group	Item	Intent to treat analysis				Per protocol analysis			
		Friedman χ^2 (df)	Kendall W	Effect size	p-value	Friedman χ^2 (df)	Kendall W	Effect size	p-value
**Intervention**	Score	79.3 (2)	0.79	Large	**<0.001***	84.2 (2)	0.86	Large	**<0.001***
	Level	19.7 (2)	0.20	Small	**<0.001***	19.7 (2)	0.20	Small	**<0.001***
**Non-intervention**	Score	28.5 (2)	0.29	Small	**<0.001***	34.9 (2)	0.36	Small	**<0.001***
	Level	6.5 (2)	0.07	Small	0.038*	5.3 (2)	0.05	Small	0.071

* Significant results as $p < 0.05$

Table (3): The pairwise comparisons in knowledge scores and levels between visits (n =100)

Item	Intervention			Nonintervention		
	Baseline visit vs. First post-intervention visit	Baseline visit vs. Second post-intervention visit	First post-intervention visit vs. Second post-intervention visit	Baseline visit vs. First post-intervention visit	Baseline visit vs. Second post-intervention visit	First post-intervention visit vs. Second post-intervention visit
Intent to treat analysis						
Score	<0.001*	<0.001*	0.069	<0.001*	0.017*	0.931
Level	0.005*	<0.015*	1.000	1.000	0.890	0.520
Per protocol analysis						
Score	<0.001*	<0.001*	0.069	<0.001*	<0.001*	0.931
Level	0.005*	0.015*	1.000	NA	NA	NA

* Significant results as $p < 0.05$. NA: not applicable (constant proportions for all visits)

Table (4): Comparison of proportions of disposing of insulin syringes or pen needles at health care facilities (n =100)

Variable	Intent to treat analysis			Per protocol analysis		
	Intervention (n = 50)	Non-intervention (n = 50)	p-value[¥]	Intervention (n = 49)	Non-intervention (n= 48)	p-value[¥]
**Baseline visit**	0 (0%)	1 (2%)	1.000	0 (0%)	1 (2%)	1.000
**First post-intervention visit**	47 (94%)	1 (2%)	**<0.001***	47 (96%)	1 (2%)	**<0.001***
**Second post-intervention visit**	47 (94%)	1 (2%)	**<0.001***	47 (96%)	1 (2%)	**<0.001***

* Significant results < 0.05. [¥] Fisher's exact test p-values

Table (5): Cochrane Q test results for the repeated measures of the proper disposal (n =100)

Treatment group	Comparison	Intent to treat analysis				Per protocol analysis			
		Improper	Proper	Q (df)	p-value	Improper	Proper	Q (df)	p-value
**Intervention**	Baseline	50 (100%)	0 (0%)	94 (2)	**<0.001***	49 (100%)	0 (0%)	94 (2)	**<0.001***
	Two-months follow up	3 (6%)	47 (94%)			2 (4%)	47 (96%)		
	Four-months follow up	3 (6%)	47 (94%)			2 (4%)	47 (96%)		
**Non-intervention**	Baseline	49 (98%)	1 (2%)	NA	NA	47 (98%)	1 (2%)	NA	NA
	Two-months follow up	49 (98%)	1 (2%)			47 (98%)	1 (2%)		
	Four-months follow up	49 (98%)	1 (2%)			47 (98%)	1 (2%)		

* Significant results < 0.05. NA: not applicable (constant proportions for all visits). df: degree of freedom

Table (6): The pairwise McNemar comparisons between visits for the proper disposal (n =100)

Variable	Intent to treat analysis		Per protocol analysis	
	**Intervention (n = 50)**	**Non-intervention (n = 50)**	**Intervention (n = 49)**	**Non-intervention (n= 48)**
**Baseline visit vs. 2**	**<0.001***	1.000	**<0.001***	1.000
**Baseline visit vs. 3**	**<0.001***	1.000	**<0.001***	1.000
**Visit 2 visit vs. 3**	1.000	1.000	1.000	1.000

* Significant results < 0.05.

Table (7): Practice scores and categories pre- and post-intervention (n =100)

Variable	Intent to treat analysis			Per protocol analysis		
	Intervention (n = 50)	Non-intervention (n = 50)	p-value	Intervention (n = 49)	Non-intervention (n= 48)	p-value
**Baseline visit**						
Score: Median (IQR)	4.4 (4-5)	4.4 (3.3-5.5)	0.585	4.4 (4-5)	4.4 (3.3-5.6)	0.671
**Categories**			0.441			0.477
Poor	30 (60%)	31 (62%)		29 (59%)	29 (60%)	
Fair	20 (40%)	17 (34%)		20 (41%)	17 (35%)	
Good	0 (0%)	2 (4%)		0 (0%)	2 (4%)	
**Median (IQR)**	1 (1-2)	1 (1-2)	0.971	1 (1-2)	1 (1-2)	0.966
**First post-intervention visit**						
Score: Median (IQR)	7.8 (6.7-8)	4.4 (3.3-5.6)	**<0.001***	7.8 (6.7-8)	4.4 (3.3-5.6)	**<0.001***
**Categories**			**<0.001***			**<0.001***
Poor	2 (4%)	30 (60%)		1 (2%)	28 (58%)	
Fair	14 (28%)	18 (36%)		14 (29%)	29 (38%)	
Good	34 (68%)	2 (4%)		34 (69%)	2 (4%)	
**Median (IQR)**	2 (2-3)	1 (1-2)	**<0.001***	2 (2-3)	1 (1-2)	**<0.001***
**Second post-intervention visit**						
Score: Median (IQR)	8 (7.8-9)	4.4 (3.3-5.6)	**<0.001***	8 (7.8-9)	4.4 (3.3-5.6)	**<0.001***
**Categories**			**<0.001***			**<0.001***
Poor	1 (2%)	28 (56%)		0 (0%)	26 (54%)	
Fair	7 (14%)	20 (40%)		7 (14%)	20 (42%)	
Good	42 (84%)	2 (4%)		42 (86%)	2 (4%)	
**Median (IQR)**	3 (2-3)	1 (1-2)	**<0.001***	3 (2-3)	1 (1-2)	**<0.001***

* Significant results < 0.05.

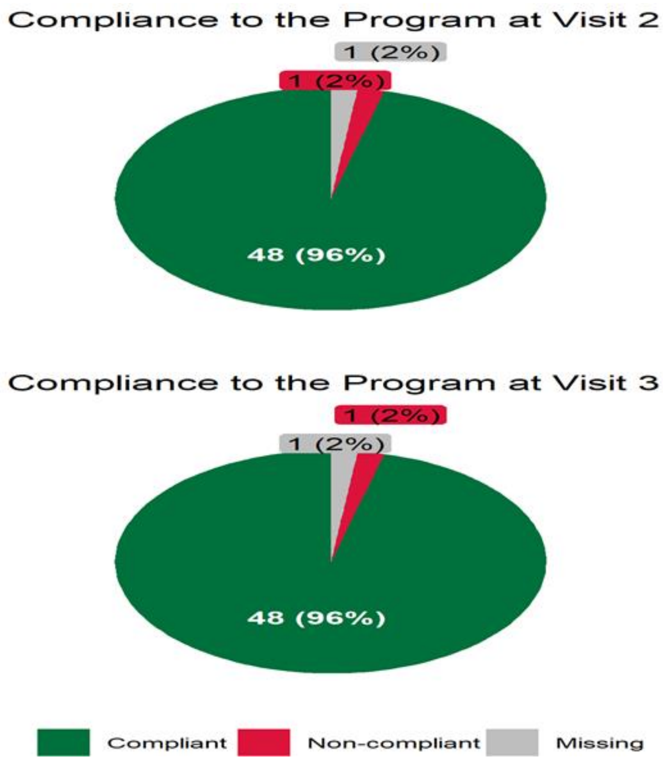

Figure (5): Compliance with the educational program
