# Supplementary material for: The effect of an educational program on the knowledge and practices of diabetic patients regarding sharps waste disposal at home
Source: Sci Rep. 2024 Dec 23;14:30590. doi: 10.1038/s41598-024-81308-y (PMC11666584; doi:10.1038/s41598-024-81308-y)
Supplement: Supplementary file 3 — Supplementary Material 3 [file 41598_2024_81308_MOESM3_ESM.pdf]

05 October 2023

To Whom It May Concern:

RE: The effect of an environmental educational program on the knowledge and practices of diabetic patients regarding sharps waste disposal at home, Alexandria, Egypt

As project manager for the Pan African Clinical Trial Registry (pactr.samrc.ac.za) database, it is my pleasure to inform you that your application to our registry has been accepted. Your unique identification number for the registry is **PACTR202310841894237**.

Please be advised that your trial is registered under an initiative within our system that allow us to capture data of trials that are already in progress or completed. As such, your trial registration may not adhere to the mandates set forth by the International Committee of Medical Journal Editors for registration requirements, and it is your duty to be transparent to any journal that may ask about the retrospective status of your registration.

Please note you are responsible for updating your trial, or for informing us of changes to your trial. Additionally, please provide us with copies of your ethical clearance letters as we must have these on file (via email or post or by uploading online) at your earliest convenience if you have not already done so.

Please do not hesitate to contact us at +27 21 938 0835 or email pactradmin@mrc.ac.za should you have any questions.

Yours faithfully,

PACTR Admin
pactr.samrc.ac.za
+27 021 938 0835

PACTR
PAN AFRICAN CLINICAL TRIALS REGISTRY
